# Colobome irien bilatéral

**DOI:** 10.11604/pamj.2018.30.1.14505

**Published:** 2018-05-02

**Authors:** Nouha Zerkaoui

**Affiliations:** 1 Université Mohammed V Souissi, Service d’Ophtalmologie A de l’Hôpital des Spécialités de Rabat, Centre Hospitalier Universitaire Rabat, Maroc

**Keywords:** Iris coloboma, fetal fissure, coloboma of the optic nerve, Iris coloboma, fetal fissure, coloboma of the optic nerve

## Abstract

Congenital ocular colobomas are due to incomplete closure of the fetal fissure during organogenesis. Ocular involvement can be variable ranging from a simple hole in the iris to a more severe involvement of the posterior pole (coloboma of the optic nerve, of the choroid, of the retina). We here report the case of a typical isolated bilateral iris coloboma. The study involved a 55-year old woman who discovered a typical inferonasal iris coloboma without involvement of the crystalline or of the posterior pole on ophthalmologic examination.

## Image en médecine

Les colobomes oculaires congénitaux sont dus à un défaut de fermeture de la fissure fœtale lors de l'organogénèse. L'atteinte oculaire pouvant être variable allant d'une simple fente irienne à une atteinte plus sévère du pôle postérieur (colobome du nerf optique, de la choroïde, de la rétine). Nous allons rapporter le cas d'un colobome typique irien bilatéral isolé. Il s'agit d'une femme de 55 ans qui lors d'un examen ophtalmologique fit la découverte d'un colobome typique irien infero-nasal sans atteinte cristallinienne ni atteinte du pôle postérieur.

**Figure 1:**
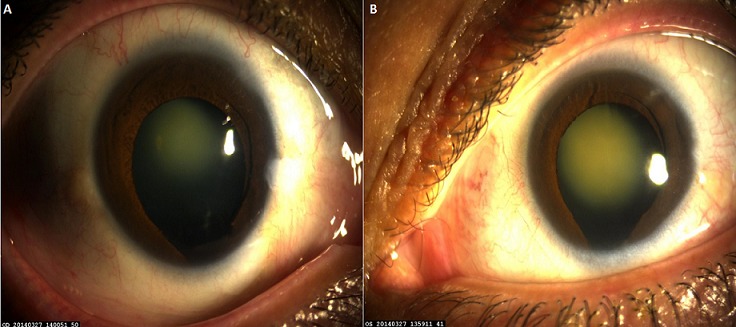
(A,B) colobome irien infero-nasal œil droit et gauche

